# Clinical implementation and evaluation of stereotactic liver radiotherapy in inspiration breath-hold using nasal high-flow therapy and surface guidance

**DOI:** 10.1093/bjr/tqae177

**Published:** 2024-09-16

**Authors:** Colien Hazelaar, Richard Canters, Kirsten Kremer, Indra Lubken, Femke Vaassen, Jeroen Buijsen, Maaike Berbée, Wouter van Elmpt

**Affiliations:** Department of Radiation Oncology (Maastro), GROW Research Institute for Oncology and Reproduction, Maastricht University Medical Centre+, Maastricht, 6229 ET, The Netherlands; Department of Radiation Oncology (Maastro), GROW Research Institute for Oncology and Reproduction, Maastricht University Medical Centre+, Maastricht, 6229 ET, The Netherlands; Department of Radiation Oncology (Maastro), GROW Research Institute for Oncology and Reproduction, Maastricht University Medical Centre+, Maastricht, 6229 ET, The Netherlands; Department of Radiation Oncology (Maastro), GROW Research Institute for Oncology and Reproduction, Maastricht University Medical Centre+, Maastricht, 6229 ET, The Netherlands; Department of Radiation Oncology (Maastro), GROW Research Institute for Oncology and Reproduction, Maastricht University Medical Centre+, Maastricht, 6229 ET, The Netherlands; Department of Radiation Oncology (Maastro), GROW Research Institute for Oncology and Reproduction, Maastricht University Medical Centre+, Maastricht, 6229 ET, The Netherlands; Department of Radiation Oncology (Maastro), GROW Research Institute for Oncology and Reproduction, Maastricht University Medical Centre+, Maastricht, 6229 ET, The Netherlands; Department of Radiation Oncology (Maastro), GROW Research Institute for Oncology and Reproduction, Maastricht University Medical Centre+, Maastricht, 6229 ET, The Netherlands

**Keywords:** hepatocellular carcinoma, liver metastases, radiotherapy, breath-hold, oxygen

## Abstract

**Objective:**

To evaluate 2 years of clinical experience with markerless breath-hold liver stereotactic radiotherapy (SBRT) using noninvasive nasal high-flow therapy (NHFT) for breath-hold prolonging and surface guidance (SGRT) for monitoring.

**Methods:**

Heated and humidified air was administered via a nasal cannula (40 L/min, 80% oxygen, 34 °C). Patients performed voluntary inspiration breath-holds with visual feedback. After a training session, 4-5 breath-hold CT scans were acquired to delineate an internal target volume (ITV) accounting for inter- and intra-breath-hold variations. Patients were treated in 3-8 fractions (7.5-20 Gy/fraction) using SGRT-controlled beam-hold. Patient setup was performed using SGRT and CBCT imaging. A posttreatment CBCT was acquired for evaluation purposes.

**Results:**

Fifteen patients started the training session and received treatment, of whom 10 completed treatment in breath-hold. Half of all 60-second CBCT scans were acquired during a single breath-hold. The average maximum breath-hold duration during treatment ranged from 47 to 108 s. Breath-hold ITV was on average 6.5 cm³/30% larger (range: 1.1-23.9 cm³/5%-95%) than the largest GTV. Free-breathing ITV based on 4DCT scans was on average 16.9 cm³/47% larger (range: −2.3 to 58.7 cm^3^/−16% to 157%) than the breath-hold ITV. The average 3D displacement vector of the area around PTV for the posttreatment CBCT scans was 5.0 mm (range: 0.7-12.9 mm).

**Conclusions:**

Liver SBRT in breath-hold using NHFT and SGRT is feasible for the majority of patients. An ITV reduction was observed compared to free-breathing treatments. To further decrease the PTV, internal anatomy-based breath-hold monitoring is desired.

**Advances in knowledge:**

Noninvasive NHFT allows for prolonged breath-holding during surface-guided liver SBRT.

## Introduction

Stereotactic radiotherapy (SBRT) is a curative treatment option for patients with oligometastases in the liver or hepatocellular carcinoma who are eligible for radical treatment, but not for surgical resection. In liver SBRT, several fractionation schemes with a total dose of 60 Gy are commonly used in the Netherlands,^1^ where for liver metastases a higher fraction dose increases the probability of local control.^2^ The fractionation scheme mainly depends on the distance of the planning target volume (PTV) to the organs at risk and the amount of healthy liver tissue that needs to be spared.^3^ However, when treating tumours in the liver in free-breathing using an internal target volume (ITV) approach, breathing motion results in a large ITV and suboptimal cone-beam CT (CBCT) image quality due to motion-induced artifacts. Also, tumour visibility is often limited on the 4DCT scan that is acquired for treatment planning. This all results in the use of large PTV margins and treatment volumes, which often result in a compromise in the dose prescription to spare healthy tissue. Some centres use implanted fiducial markers, possibly combined with gating or tracking techniques, to help guide tumour delineation and patient positioning, and decrease treatment volumes.^4–11^ However, this requires an invasive procedure with associated risks and is therefore not preferred in all centres, including our centre. Alternatively, MR-guided treatment systems are being used,^12–14^ but these are currently only available in selected centres.

A possible method to improve imaging and treatment quality is to scan and treat patients in breath-hold. Since breath-hold limits the main motion component of the liver, the ITV would be reduced and therefore also the PTV when considering the same ITV-PTV margin. Previously, this has been shown to be dosimetrically and clinically beneficial due to a reduction of normal tissue doses, possibly allowing for a higher fraction dose and therefore a higher chance of local control.^15–18^ However, when treating patients in breath-hold, treatment times usually become longer because patients need multiple breath-holds and time in between the breath-holds, during which irradiation is halted.^19^ To increase treatment efficiency, we therefore aimed to prolong the breath-hold. In a previous study, the feasibility of additional oxygen supply using nasal high-flow therapy (NHFT) during breath-hold treatment was investigated for locally advanced lung cancer patients.^20^ It was found that NHFT prolonged the breath-hold by a mean factor of 2.1 (range 1.1-3.9), from, on average, 39-78 s, and the subjective tolerance to the treatment was good. Based on this, we decided to apply this technique for liver SBRT.

The aim was to clinically implement a treatment protocol for markerless liver SBRT in breath-hold using additional noninvasive oxygen supply using NHFT to obtain a prolonged voluntary breath-hold and visual guidance by a surface-guided radiotherapy (SGRT) system. In this study, we evaluate the first 2 years of clinical experience with this treatment protocol.

## Methods

**Figure 1.**
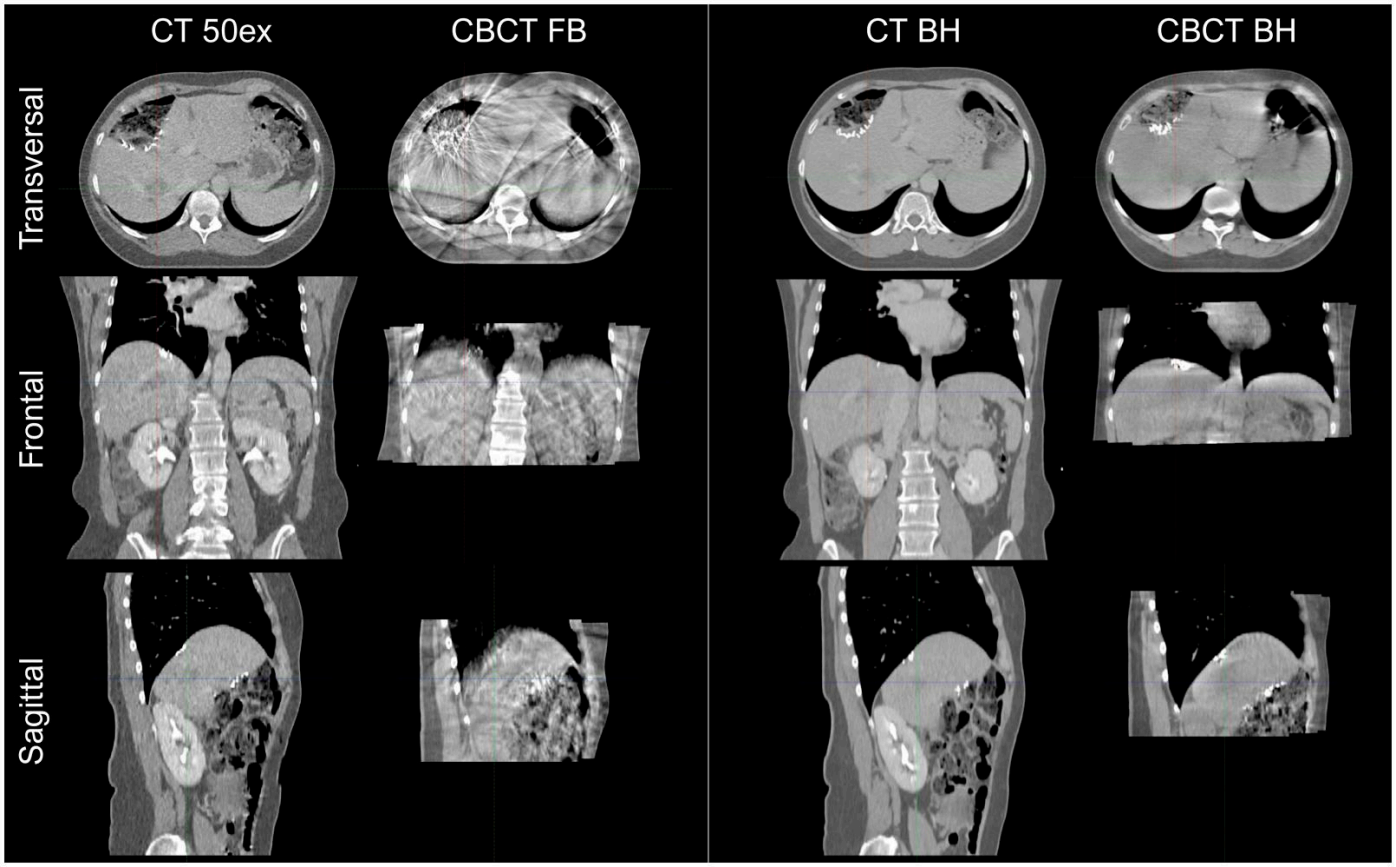
Example of a free-breathing (FB) planning CT scan, which is the 50% exhale phase of a 4DCT scan, a cone-beam CT (CBCT) scan acquired in free-breathing, a breath-hold (BH) CT scan, and a breath-hold CBCT scan. These scans were acquired of a pilot patient, in which both CBCT scans were acquired during the same treatment fraction. In the free-breathing CBCT scan, blurring and artifacts are observed due to breathing motion, which disappeared for the breath-hold CBCT scan.

After completion of a pilot study that showed the feasibility of inspiration breath-hold (IBH) in liver SBRT patients as well as a visually improved image quality due to the disappearance of artifacts caused by breathing motion (Figure 1), the breath-hold protocol was developed and clinically implemented in June 2020. Until June 2022, 17 patients were eligible to start with the liver SBRT procedure.

### Devices and treatment preparation

The NHFT was delivered using the Airvo-Optiflow system (Fisher & Paykel Healthcare Ltd, Auckland, New Zealand), which is a noninvasive system that delivers a high flow of heated and humidified air via a nasal cannula. A flow of 40 L/min with 80%-oxygen and a temperature of 34 °C were used, similar to our previous study.^20^ For SGRT, the Sentinel and Catalyst HD systems (C-RAD, Uppsala, Sweden) were used in the CT and treatment room, respectively. Voluntary breath-holds were performed and visual coaching was provided to the patient via virtual reality goggles, through which patients could observe their breathing amplitude, baseline level (ie, average end-expiration level), and gating window. This breathing signal was measured at a point of 4 cm diameter defined on the patient’s surface at the location of the xiphoid process.

A training session at the CT scanner was performed to familiarize the patient with the NHFT and SGRT devices, to determine whether the patient was suitable for treatment in breath-hold and if so, to define the voluntary breath-hold level and average breath-hold duration with NHFT. Patients were deemed suitable for treatment in breath-hold if they were able to understand the procedure, able to perform breath-holds of at least 20 seconds, and if the breath-holds were stable, which was assessed visually based on the SGRT signal. Otherwise, patients were treated in free-breathing. During the training session, patients were positioned in treatment position, that is, supine on a lungboard (MacroMedics BV, Moordrecht, The Netherlands) with, if possible, the arms above the head, without the use of immobilization devices, and connected to the NHFT and SGRT systems. An IBH level that felt comfortable to the patient (ie, a moderate deep IBH to allow for relatively long and stable breath-holds) was first defined without visual guidance, after which this was confirmed with visual guidance. During CT and treatment, a 2-mm gating window was used based on this breath-hold level. Patients were instructed to keep their breath-hold within the gating window as long as possible.

**Figure 2.**
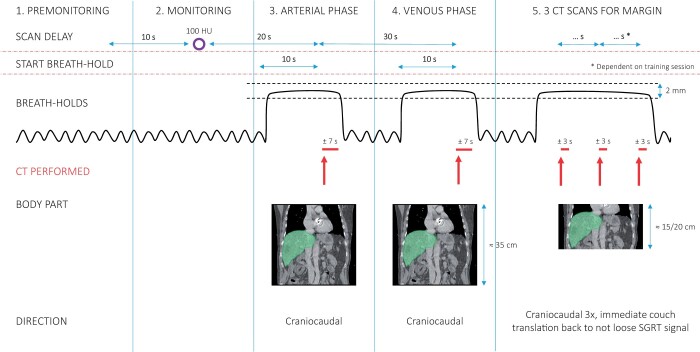
Contrast-enhanced breath-hold CT protocol. In total 4-5 CT scans, depending on the average breath-hold duration during the training session, were acquired in breath-hold.

If the patient successfully completed the training session, the CT procedure followed immediately. The contrast-enhanced breath-hold CT protocol (Figure 2) consisted of administering intravenous contrast agents (flow 3 mL/s, 150 mL Ultravist-300, and 30 mL NaCl), followed by multiple breath-hold CT scans: an arterial phase CT, venous phase CT, and 2-3 CT scans acquired at the beginning, middle, and end of a single breath-hold, which is to estimate both inter- and intra-breath-hold variations to delineate a breath-hold ITV. Afterwards, a respiratory-correlated 4DCT scan was acquired for backup purposes in case free-breathing treatment was needed. The combined training and CT session was performed within a 90-minute time slot.

### Treatment planning

Treatment planning was performed on the venous phase CT scan in our treatment planning system ([TPS], Eclipse version 15.5 or 16.1, Varian, Palo Alto, CA, USA). The gross tumour volume (GTV) was delineated on the venous phase CT and the 2-3 intra-breath-hold CT scans, and the breath-hold ITV was created by merging these GTVs. The PTV was then defined as ITV+1 cm isotropic margin. This margin was based on our free-breathing liver SBRT protocol where the image quality was poor. Although improvements were expected with the breath-hold protocol, it was decided to use this margin until an evaluation could be performed, also because only external surface and no internal breath-hold monitoring was performed during treatment. The benefit for the patient of breath-hold compared to treatment in free-breathing was, therefore, the reduction in ITV, and therefore also a smaller treatment volume. The prescription dose was 60 Gy delivered in 3, 5, 8, or 12 fractions, depending on what was achievable concerning OAR doses (using the constraints reported by Méndez Romero et al^1^), or 3 × 8 Gy for patients with oligo progression who received immunotherapy. The goal was to cover 99% of the PTV with 100% of the prescribed dose (ie, 60 or 24 Gy), with a maximum dose of up to 120%. However, sparing of OARs was prioritized over PTV coverage, that is, the PTV could be underdosed in case of PTV-OAR overlap (up to 75% of the prescribed dose). Patients were treated on a TrueBeam (version 2.7, Varian), typically using 2-3 half or partial volumetric modulated arc therapy arcs, mainly using 6 MV FFF.

Patient QA was performed before the first treatment fraction using an Octavius 4D phantom with Standard Top and Octavius SRS 1000 detector (PTW, Freiburg, Germany).

### Treatment delivery

The first treatment fraction was scheduled 5 working days after the planning CT scan. For the first fraction, a time slot of 40 minutes was scheduled, and for subsequent fractions 30 minutes. During each fraction, the patient was connected to the NHFT and SGRT devices and positioned on a 6-degree-of-freedom (DoF) couch using the laser lines, followed by finetuning using the SGRT system. A new baseline of the breathing signal was calculated before imaging and treatment and the gating window was automatically set at a relative distance from this baseline, similar to during CT acquisition, and a new reference image of the patient’s surface, to which the live image is compared for motion monitoring, was acquired during a short breath-hold. Pretreatment imaging consisted of a half-fan CBCT scan with a 60-second acquisition time acquired in breath-hold, where the beam was manually switched on and off when the patient was inside or outside the gating window, respectively. An automatic 4-DoF CBCT-CT match was performed based on the liver structure delineated on the planning CT scan plus a 1-cm margin, with manual corrections if needed, for example, with the focus on the area around the PTV or OARs. During treatment delivery, automatic beam control was used based on whether the actual breathing signal was within the 2 mm gating window, as well as motion detected by continuously comparing the actual patient surface with the reference surface, using a surface tolerance of 8-10 mm (10 mm was a standard value used for the first 2 patients, this was decreased to 8 mm for subsequent patients unless a medical physicist decided to increase the tolerance) and target tolerance (based on the isocentre position retrieved from the treatment plan) of 4-5 mm. After treatment, an additional CBCT scan was acquired for evaluation purposes.

### Evaluation

The breath-hold duration with the use of NHFT and the number of breath-holds that were performed during the CBCT acquisition and treatment phase (ie, all breath-holds that were performed, not only the breath-holds where the treatment beam was on) were calculated in Matlab (version R2021b, MathWorks, Natick, MA, USA) based on the breathing signal of the SGRT. However, the breathing signal was not available for all CBCT scans: for patients with targets located laterally, a couch translation (Center Couch) procedure was performed during CBCT acquisition. Because manual shifts of the breathing point of the SGRT system had to be made, the breathing signal could not be saved for these CBCT scans. Therefore, for these cases, the number of folders the CBCT projection data consisted of was used instead for determining the number of breath-holds. The overall treatment time from CBCT before treatment to CBCT after treatment, or end of treatment delivery in case no CBCT after treatment was acquired, was extracted from the record and verify system (Offline Review, ARIA, Varian).

For the breath-hold CT scans, the variation in GTV centroid position was determined in the TPS to evaluate inter- and intra-breath-hold variation. However, this includes both breath-hold and delineation variations, and therefore the volumes of the GTVs were also assessed. Besides, free-breathing ITVs were determined, based on the 4DCT scans, to compare to the breath-hold ITVs. Due to limited tumour visibility, for most patients 6-DoF registrations based on the area around the PTV were performed between the planning CT scan and all phases of the 4DCT scan. The breath-hold GTV was then rigidly transferred to all phases of the 4DCT scan, and the free-breathing ITV was created by merging these GTVs.

To evaluate the breath-hold reproducibility at the time of treatment, the posttreatment CBCT scan was matched to the planning CT scan using the same 4-DoF match protocol as used clinically. For the patients with >1 posttreatment CBCT, the systematic error (Σ) was calculated by averaging the match results for the left-right (LR), superior-inferior (SI), and anterior-posterior (AP) directions per patient followed by computation of the standard deviation across the patients, and the random error (σ) by calculating the standard deviation per patient followed by computation of the root mean square across the patients.

## Results

**Figure 3.**
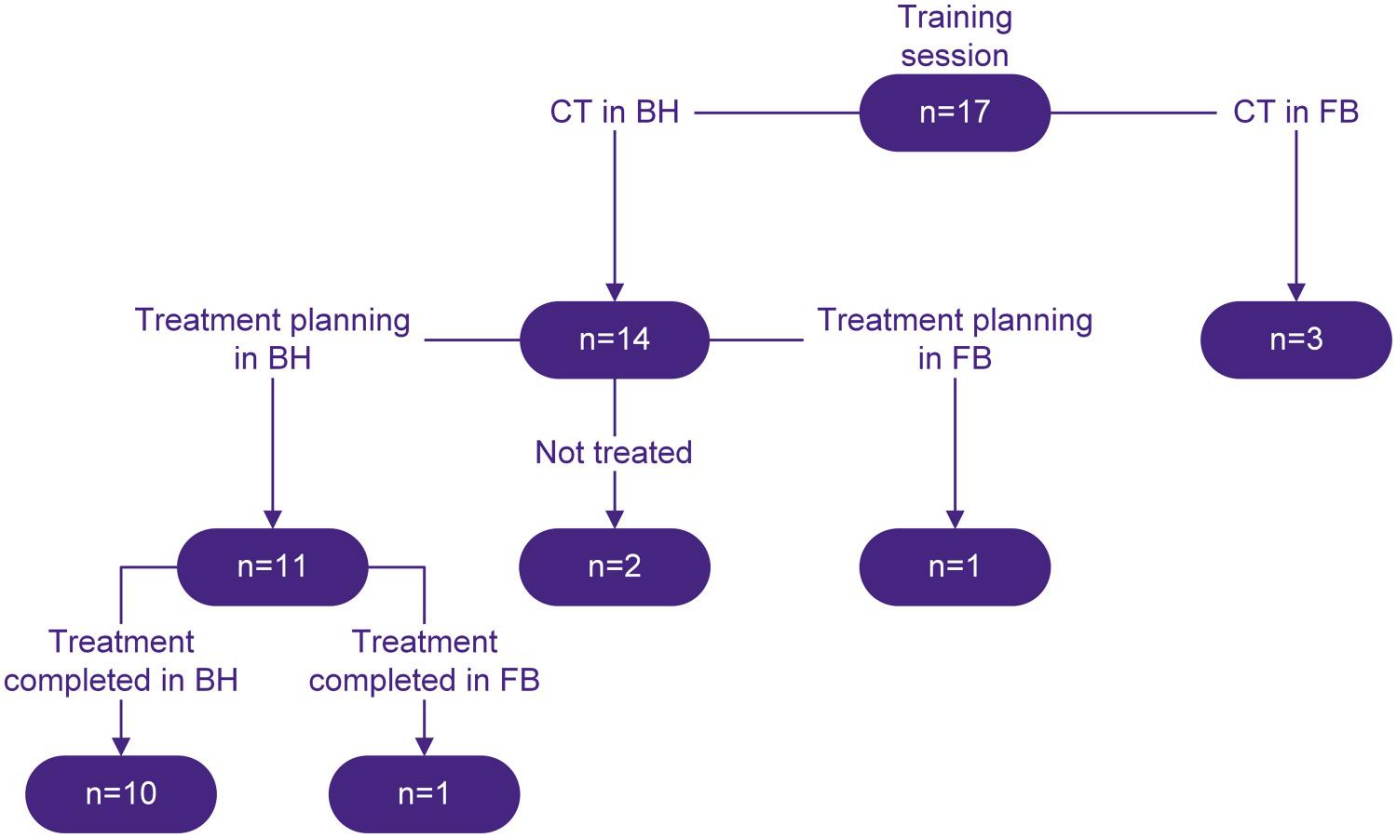
Flowchart showing the number of breath-hold (BH) patients and patients who continued in free-breathing (FB) for each step of the workflow.

Of the 17 patients who started with the training session, 10 completed the treatment in breath-hold (Figure 3). Two patients did not receive radiotherapy because of an increase in metastases, and 5 patients continued at some point in free-breathing. Reasons for continuing the CT in free-breathing were inability to understand the procedure or position the arm above the head for a longer time. One patient was treated in free-breathing (using the backup 4DCT scan) because of large deviations in the GTV position (∼2 cm) observed on the breath-hold CT scans (for this patient, it was difficult to maintain a stable breath-hold within the gating window). Patient 5 continued in free-breathing after the first fraction because the patient was not able to perform stable breath-holds due to a nontreatment-related medical condition. Patient and treatment characteristics of all patients who started treatment in breath-hold are shown in Table 1. The dose-volume histogram (DVH) parameter values for each patient based on the constraints used for the different fractionation schemes are shown in Table S1. All OAR constraints were fulfilled, however, for some patients, it was required to sacrifice PTV coverage to fulfil the OAR constraints. No severe or life-threatening (acute) toxicity (CTCAE grade ≥3) was observed. Mild or moderate symptoms that were reported included fatigue, nausea, and chest/thoracic wall pain.

**Table 1.** Patient and treatment characteristics.

Patient	Sex	Age (y)	PTV (cm^3^)	Dose prescription (fractions × Gy)	Energy	Maximum dose rate (MU/min)	MU/arc	Median number of BHs performed during CBCT imaging (range)	Median number of BHs performed during treatment (range)
1	F	55	58.7	5 × 12	6X-FFF	1400	965/945/865	1 (1-5)^f^	3 (2-8)
2	F	80	95.5	3 × 20	10X-FFF	1600	1832/1614/1514	1 (1-2)	7 (5-10)
3	M	73	177.4	8 × 7.5	10X-FFF	2400	1000/1165^d^	2 (1-4)	6.5 (3-19)
							960/1118		
4^a^	F	72	38.9	5 × 12	10X-FFF^d^	2400^d^	1936/1912^d^	1 (1-2)	6 (4-7)
					6X-FFF	1400	1312/1717		
5	M	73	182.4	5 × 12	6X-FFF	1400	1325/1524	2.5 (1-4)	19^g^
6	M	67	190.9	5 × 12	6X-FFF	1400	1218/1497	2 (1-3)	8 (4-13)
7^a^	M	80	242.8	8 × 7.5	6X-FFF	1400	817/859	2 (1-4)	4 (3-8)
8	M	68	91.2	5 × 12	6X-FFF	1400	1776/1824	1 (1-5)	5 (3-5)
			18.5^b^						
9	M	76	225.2^c^	3 × 8	6X-FFF	1400	1232/1136	2 (1-4)	5 (4-14)
10	M	32	72.9	3 × 20	6X-FFF	1400	3065/3065	1.5 (1-4)	9 (6-17)
11	M	70	151.7	3 × 8	6X-FFF	1400	313/783/723/305^e^	2.5 (1-3)	5 (4-6)

Abbreviations: PTV=planning target volume, MU=monitor unit, BH=breath-hold, CBCT=cone-beam CT, F=female, M=male, FFF=flattening filter free.

a These patients did not have a metastasis in the liver itself but close to the liver: patient 4 had a metastasis next to/below the liver, and patient 7 between the liver and thoracic wall.

b Two PTVs treated in a single plan, of which one was located within the liver and one between the ribs.

c This PTV consisted of multiple GTVs.

d After the first fraction, these patients were treated using a new treatment plan.

e This patient was positioned with the arms alongside the body. More treatment arcs were used to avoid irradiation through the arms.

f CBCT information of one fraction was missing and is therefore not included.

g This patient received only one fraction in breath-hold.

**Figure 4.**
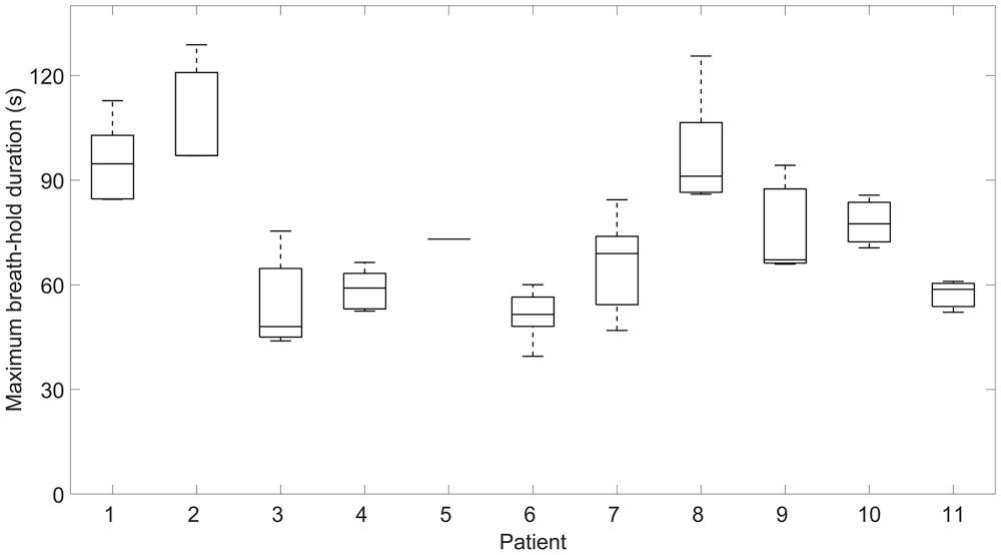
Boxplot showing for each patient the longest breath-hold per fraction during the treatment phase.

The maximum breath-hold duration during treatment is visualized in Figure 4. The average maximum breath-hold duration per patient ranged from 47 to 108 s. For this analysis, breath-holds during CBCT imaging were excluded because the breathing signal of the CBCT imaging was not available for all patients. Furthermore, a breath-hold of >60 s was usually not required for CBCT imaging. However, it was observed that for some fractions, the maximum breath-hold duration was longer during CBCT imaging compared to treatment.

The median number of breath-holds performed during CBCT imaging and the treatment phase are shown for each patient in Table 1. For the 100 60-second CBCT scans, the average number of breath-holds per CBCT scan was 1.8 ± 1.0 (range: 1-5). Half of all CBCT scans were acquired during a single breath-hold. For all patients, at least one CBCT scan could be acquired during a single breath-hold.

**Figure 5.**
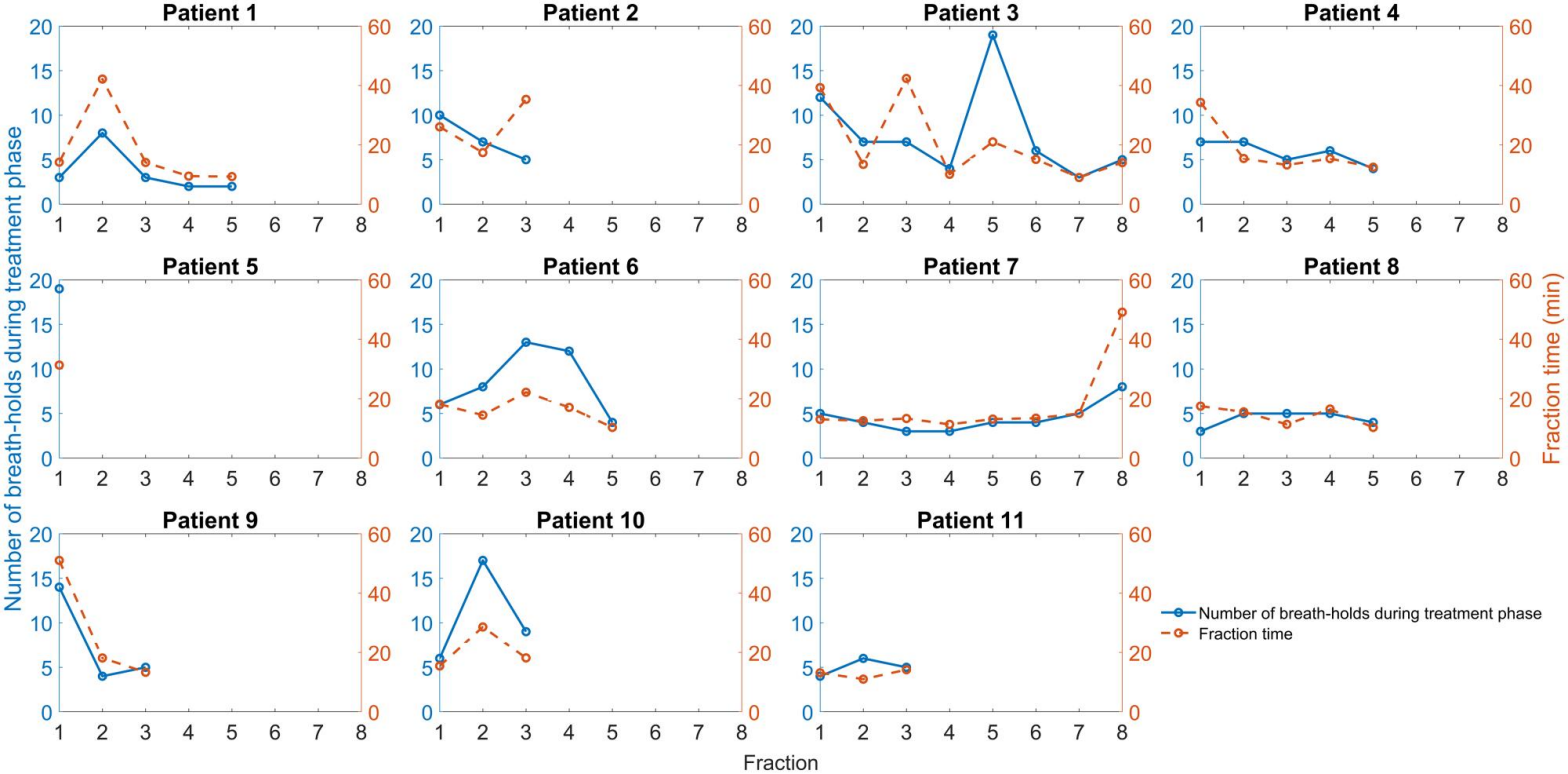
Number of breath-holds performed during the treatment phase of each fraction (left axis; solid, blue) together with the time from the first cone-beam CT (CBCT) until the posttreatment CBCT scan or end of treatment if no CBCT after treatment was acquired (right axis; dashed, orange).

The average time from CBCT before treatment to CBCT after treatment (or end of treatment) was 19 ± 11 minutes (range: 9-51 min). No obvious reduction in the number of breath-holds that were performed or fraction time was observed for later treatments, both within and between patients, which would have indicated a learning curve (Figure 5). However, some outliers were present, which were mainly due to technical issues related to the SGRT system.

**Figure 6.**
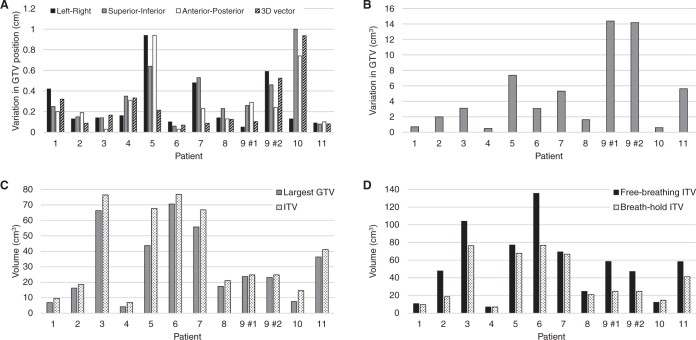
(A) Variation in GTV centroid position on the breath-hold CT scans for the individual patients and directions, (B) variation in GTV delineated on the breath-hold CT scans, (C) largest GTV compared to breath-hold ITV, and (D) breath-hold ITV compared to free-breathing ITV. For patient 9, the PTV consisted of multiple GTV/ITVs, of which 2 GTVs were delineated on all scans and therefore analysed separately. Abbreviations: GTV = gross tumour volume, ITV=internal target volume, PTV=planning target volume.

The difference in centroid position of the GTVs delineated on the breath-hold CT scans was on average 3 ± 3 mm for all directions (range: 0-10 mm, Figure 6A). The median volume difference of the GTVs was 3.1 cm^3^ (range: 0.5-14.4 cm^3^), which corresponds to a median volume difference of 11% of the largest GTV per patient (range: 4%-61%, Figure 6B). For all patients with a GTV difference >4 cm^3^, it was noted that the tumour visibility decreased over time due to the washout of contrast, resulting in smaller volumes on subsequent CT scans. When comparing the largest GTV to the breath-hold ITV, it was found that the breath-hold ITV was on average 6.5 cm^3^/30% larger (range: 1.1-23.9 cm^3^/5%-95%) than the GTV (Figure 6C). Free-breathing ITVs were on average 16.9 cm^3^/47% larger (range: −2.3 to 58.7 cm^3^/−16% to 157%) than the breath-hold ITVs (Figure 6D).

**Figure 7.**
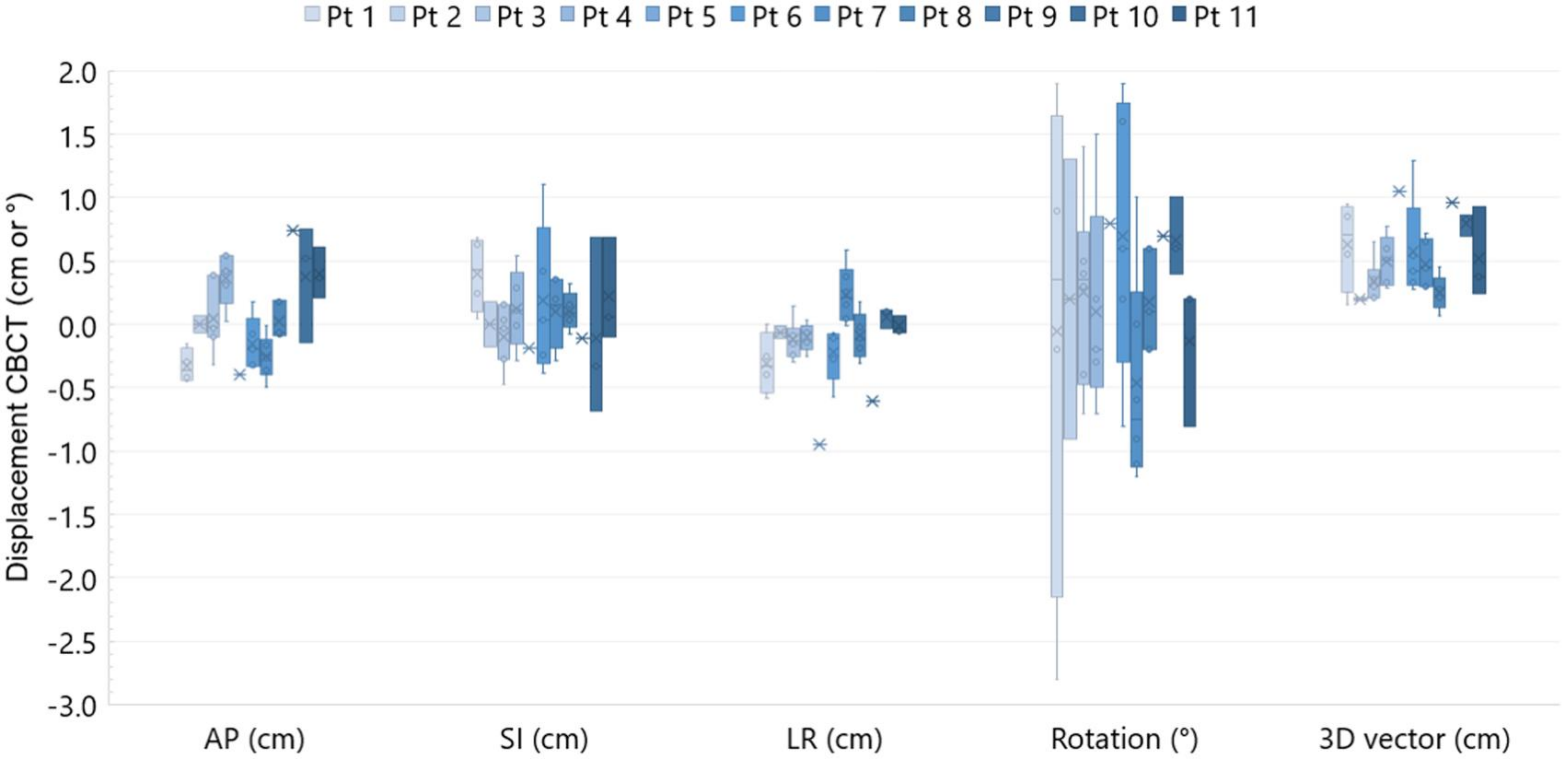
Boxplot visualizing the offset of the posttreatment CBCT scans compared to the planning CT scan for the individual patients and directions, as well as the 3D vector of the displacement (excluding the rotational component). For patient 9, one CBCT scan was not included because of difficulties with matching. Abbreviations: AP=anterior-posterior, SI=superior-inferior, LR=left-right, CBCT=cone-beam CT.

In total 43 CBCT scans acquired after treatment were registered to the planning CT scan. The 3D displacement vector was on average 5.0 mm (range: 0.7-12.9 mm) and for all but 2 scans less than our 1 cm PTV margin. All but one of the translational offsets were within 1 cm, the rotational deviation was up to 2.8° (Figure 7). Based on the posttreatment CBCT-CT registrations, the systematic error was Σ_LR_ = 1.6, Σ_SI_ = 1.6, Σ_AP_ = 2.8 mm, and the random error σ_LR_ = 1.6, σ_SI_ = 4.0, σ_AP_ = 2.4 mm.

## Discussion

In this study, we successfully implemented a markerless breath-hold liver SBRT protocol using NHFT for prolonging the breath-holds and SGRT, which resulted in visually high-quality CT and CBCT images. Although the number of patients receiving liver SBRT was limited, evaluation of the 2 years after clinical implementation showed that for 5 out of the 15 patients who started with the training session and received treatment, the treatment procedure was at some point continued in free-breathing, indicating that our breath-hold protocol is feasible for ∼67% of the patients. This compliance is also within the range of other published studies, where reported compliance rates range between 60% and 95%.^19^^,^^21–26^ Although for most patients, treatment in breath-hold resulted in smaller ITV and therefore smaller treatment volumes compared to free-breathing, which is likely to result in a dosimetric benefit,^15^^,^^17^^,^^18^ this was not the case for all patients. Two patients showed no or only a minor reduction (in percentage; <10%), which was for the 2 patients that did not have metastasis in, but close to the liver (ie, patients 4 [0.0%] and 7 [3.7%]). For patient 10, the breath-hold ITV was larger compared to the free-breathing ITV due to large breath-hold variations. To select patients that benefit most from treatment in breath-hold, an additional selection criterion will therefore be considered. In addition, our current clinical procedure is to delineate the GTV on all breath-hold CT scans, however, it was noted that for some patients, the volumes became smaller on subsequent CT scans due to the washout of contrast. To overcome this decrease in volume, the GTV could be delineated on the first CT scan and copied to the subsequent scans after performing accurate image registration.

A frequently described method for establishing breath-holds in liver SBRT is the use of the Active Breathing Coordinator (ABC, Elekta Solutions AB, Stockholm, Sweden).^21^^,^^23–25^^,^^27–35^ With this technique, breath-holds of 10-30 s are usually performed.^21^^,^^24^^,^^25^^,^^28–32^ Zhong et al^33^ modified the ABC system to allow for an oxygen flow to prolong breath-holds, resulting in an average maximum end-inhale breath-hold duration of 65 s after training. As shown in studies previously performed in our centre, NHFT prolonged the breath-hold by a factor of 2,^20^^,^^36^ which resulted in the current study in a maximum breath-hold duration of up to 129 s (Figure 4). Vakaet et al^37^ investigated different oxygenation devices and parameters and achieved an average breath-hold duration of 3 minutes for breast cancer patients using a combination of voluntary hyperventilation and oxygenation using NHFT, indicating possibilities for further optimizing NHFT protocols. Alternative techniques for breath-hold prolonging have been described that allow for breath-holds of >5 minutes, such as high-frequency percussive ventilation^38^ and the use of a mechanical ventilator for preoxygenation, mechanical hyperventilation, and hypocapnia.^39–41^ However, these techniques are generally more difficult to implement clinically.

Some outliers in treatment time were observed, and the reason for this was often related to issues with the SGRT system, for example, loss of breathing signal due to blockage of the SGRT cameras when the gantry rotated, resulting in automatic beam holds that would not necessarily be required. For the number of breath-holds that were performed during the treatment phase, all breath-holds were included, that is, even if there was no beam on during that breath-hold. In case technical issues occurred, often more breath-holds were performed since the beam was controlled by the SGRT system. For the CBCT acquisition, the beam was manually turned on/off, which could have resulted in inter- and intra-observer variability and might explain why for some patients the maximum breath-hold time was longer during CBCT than during the treatment phase. When the maximum breath-hold time was longer during CBCT than during treatment, the breath-hold was often also longer for the CBCT scan afterwards, indicating that exhaustion was not necessarily the issue.

The CBCT scan after treatment showed an average 3D vector displacement of 5.0 mm with a maximum of 12.9 mm. Although our inter-breath-hold variations appear to be larger compared to studies using the ABC system, a direct comparison is difficult due to differences in methodologies, criteria for selecting patients, and reporting of different metrics.^42^ Studies using ABC reported inter- and/or intra-breath-hold variations <5 mm for most patients.^21^^,^^23^^,^^27–34^ However, variations >10 mm were also observed,^34^^,^^35^ similar to our study. In our centre, all liver SBRT patients are treated in IBH because it is usually more comfortable and therefore easier to maintain compared to expiration breath-hold (EBH). Besides, this was a practical decision due to our experience with IBH. However, EBHs have been shown to be more reproducible compared to IBHs,^43^ although this might differ between patients.^32^ Stick et al^22^ assessed inter- and intra-breath-hold variations of 20-second voluntary DIBHs with visual guidance using the Real-time Position Management (RPM) system (Varian). They report a similar inter-breath-hold variability based on 3 consecutive CT scans (median 3 mm, range 0-9 mm) and posttreatment CBCT scans (median 3 mm, range 1-14 mm) as in our study. The maximum difference in marker position (superior-inferior direction) on planar kV images during a single fraction was 7-13 mm and within a single breath-hold 3-10 mm despite the gating window of 2.2-3.0 mm. Naumann et al,^44^ who also used SGRT to monitor DIBHs, reported intrafractional differences ranging from −27.8 to 28.3 mm, with an interquartile range of 2.8 mm. As also described in the literature, the correlation between the patient’s surface and internal anatomy might be limited.^22^^,^^45^^,^^46^ Currently, we use a 1-cm PTV margin on top of the breath-hold ITV that is created from the GTVs delineated on the multiple CT scans. When applying the Van Herk margin formula^47^ as used in the systematic review by Sharma et al,^42^ that is, margin = 2.5Σ + 0.4σ, our required margin is approximated to be LR = 4.7, SI = 5.7, and AP = 7.8 mm. However, this is an approximation and based on a limited number of patients and CBCT scans. Before decreasing the PTV margin, we prefer the implementation of breath-hold monitoring based on internal anatomy, especially because of drifts due to gradual lung deflation that have been shown during prolonged breath-holds.^41^ Internal monitoring can be done on a standard linear accelerator using kV imaging during treatment delivery^22^^,^^35^^,^^45^^,^^48^ or using dedicated solutions such as an additional kV source-detector system, MR-guided treatment approaches,^12–14^^,^^46^ or the use of ultrasound,^27^ which is currently not yet commercially available.

## Conclusion

Liver SBRT in breath-hold using NHFT to prolong the breath-holds and SGRT is feasible for the majority of patients. An ITV reduction was observed compared to free-breathing treatments, however, to further decrease the PTV, internal anatomy-based inter- and intra-breath-hold monitoring is desired.

## Supplementary Material

tqae177_Supplementary_Data
