# Supplementary material for: Clinical implementation and evaluation of stereotactic liver radiotherapy in inspiration breath-hold using nasal high-flow therapy and surface guidance
Source: Br J Radiol. 2024 Sep 16;97(1164):1950–8. doi: 10.1093/bjr/tqae177 (PMC11573126; doi:10.1093/bjr/tqae177)
Supplement: tqae177_Supplementary_Data [file tqae177_supplementary_data.docx]

Supplementary material

*Clinical implementation and evaluation of stereotactic liver radiotherapy in inspiration breath-hold using nasal high flow therapy and surface guidance*

**Supplementary Table 1:** Dose-volume histogram (DVH) parameter values for each patient based on the constraints used for the different fractionation schemes. Patients 3 and 4 were treated using a new treatment plan after the first fraction, therefore the values for both treatment plans are shown. For some patients, sparing of organs at risk (OARs) was prioritized over planning target volume (PTV) coverage, which is shown by PTVs that were cropped to specific OARs (according to our clinical protocol, more information provided below the tables of the relevant patients) and/or PTV D99% values below 60 Gy (for the patients receiving a total dose of 60 Gy).

| **Patient 1 (5 × 12 Gy)** |  |  | **Patient 2 (3 × 20 Gy)** |  |
| --- | --- | --- | --- | --- |
| **DVH parameter** | **Value** |  | **DVH parameter** | **Value** |
| PTV D99% | 60.0 Gy |  | PTV D99% | 49.3 Gy^a^ |
| PTV Dmax | 68.0 Gy |  | PTV Dmax | 70.7 Gy |
| PTV Dmean | 63.2 Gy |  | PTV Dmean | 63.5 Gy |
| Liver-ITV V18Gy | 207.6 cm^3^ |  | Liver-ITV V15Gy | 284.5 cm^3^ |
| Spinal Canal Dmax | 5.3 Gy |  | Spinal Canal Dmax | 12.8 Gy |
| Kidneys V15Gy | 0.0% |  | Kidneys V15Gy | 0.0% |
| Esophagus Dmax | 16.4 Gy |  | Esophagus Dmax | 16.4 Gy |
| Stomach Dmax | 17.6 Gy |  | Stomach Dmax | 10.4 Gy |
| Heart Dmax | 27.5 Gy |  | Heart Dmax | 23.2 Gy |
|  |  |  | ^a^ The PTV contained lung tissue; with a density override of the lung tissue, which is done for plan optimization, the D99% is 60 Gy. | |
| **Patient 3 (8 × 7.5 Gy)** |  |  | **Patient 4 (5 × 12 Gy)** |  |
| **DVH parameter** | **Value** |  | **DVH parameter** | **Value** |
| PTV (cropped^a^) D99% | 56.7 / 54.4 Gy |  | PTV (cropped^a^) D99% | 60.0 / 60.0 Gy |
| PTV (cropped) Dmax | 69.5 / 66.7 Gy |  | PTV (cropped) Dmax | 68.4 / 68.0 Gy |
| PTV (cropped) Dmean | 63.6 / 61.0 Gy |  | PTV (cropped) Dmean | 64.4 / 64.4 Gy |
| Liver-ITV V21.6Gy | 354.5 / 333.5 cm^3^ |  | Liver-ITV V18Gy | 96.0 / 92.0 cm^3^ |
| Spinal Canal Dmax | 20.6 / 19.8 Gy |  | Spinal Canal Dmax | 21.2 / 18.9 Gy |
| Esophagus Dmax | 9.6 / 9.2 Gy |  | Kidneys V15Gy | 4.2 / 4.3% |
| Stomach Dmax | 13.2 / 12.7 Gy |  | Kidney_R V18Gy | 7.6 / 7.8% |
| Bowel Dmax | 43.3 / 41.5 Gy |  | Esophagus Dmax | 5.5 / 4.8 Gy |
| Bowel V32.8Gy | 2.6 / 1.9 cm^3^ |  | Stomach Dmax | 14.4 / 17.8 Gy |
| Heart Dmax | 1.3 / 1.2 Gy |  | Bowel Dmax | 30.6 / 30.0 Gy |
| Thoracic Wall Dmax | 105.5 / 101.3% |  | Bowel V26Gy | 0.2 / 0.3 cm^3^ |
| ^a^ The PTV was cropped to exclude the thoracic wall and bowel tissue. | |  | Heart Dmax | 0.5 / 0.7 Gy |
|  |  |  | ^a^ The PTV was cropped to exclude the thoracic wall. | |

| **Patient 5 (5 × 12 Gy)** |  |  | **Patient 6 (5 × 12 Gy)** |  |
| --- | --- | --- | --- | --- |
| **DVH parameter** | **Value** |  | **DVH parameter** | **Value** |
| PTV (cropped^a^) D99% | 50.0 Gy^b^ |  | PTV (cropped^a^) D99% | 60.0 Gy |
| PTV (cropped) Dmax | 68.0 Gy |  | PTV (cropped) Dmax | 67.1 Gy |
| PTV (cropped) Dmean | 62.8 Gy |  | PTV (cropped) Dmean | 63.9 Gy |
| Liver-ITV V18Gy | 437.9 cm^3^ |  | Liver-ITV V18Gy | 567.2 cm^3^ |
| Spinal Canal Dmax | 4.8 Gy |  | Spinal Canal Dmax | 6.4 Gy |
| Kidneys V15Gy | 0.0% |  | Kidneys V15Gy | 0.0% |
| Esophagus Dmax | 10.2 Gy |  | Esophagus Dmax | 5.5 Gy |
| Stomach Dmax | 11.7 Gy |  | Stomach Dmax | 15.1 Gy |
| Bowel Dmax | 16.2 Gy |  | Heart Dmax | 3.2 Gy |
| Heart Dmax | 8.8 Gy |  | ^a^ The PTV was cropped to exclude the thoracic wall. | |
| ^a^ The PTV was cropped to exclude the thoracic wall. ^b^ The PTV contained lung tissue; with a density override of the lung tissue, which is done for plan optimization, the D99% is 60 Gy. | |  |  |  |
| **Patient 7 (8 × 7.5 Gy)** |  |  | **Patient 8 (5 × 12 Gy)** |  |
| **DVH parameter** | **Value** |  | **DVH parameter** | **Value** |
| PTV D99% | 44.6 Gy^a^ |  | PTV D99% | 19.1 Gy^a^ |
| PTV Dmax | 71.1 Gy |  | PTV Dmax | 72.0 Gy |
| PTV Dmean | 61.6 Gy |  | PTV Dmean | 59.1 Gy |
| Liver-ITV V21.6Gy | 274.6 cm^3^ |  | Liver-ITV V18Gy | 477.0 cm^3^ |
| Spinal Canal Dmax | 3.3 Gy |  | Spinal Canal Dmax | 14.1 Gy |
| Kidneys V20Gy | 0.0% |  | Kidneys V15Gy | 0.0% |
| Esophagus Dmax | 2.4 Gy |  | Esophagus Dmax | 26.8 Gy |
| Stomach Dmax | 15.2 Gy |  | Stomach Dmax | 2.5 Gy |
| Bowel Dmax | 9.4 Gy |  | Bowel Dmax | 31.5 Gy |
| Heart Dmax | 1.2 Gy |  | Bowel V26Gy | 3.1 cm^3^ |
| Thoracic Wall Dmax | 105.5 % |  | Heart Dmax | 36.4 Gy |
| ^a^ Lower coverage close to the patient’s surface; this PTV had to be cropped by 3 mm to the body contour for plan optimization. | | | ^a^ This PTV overlapped with the heart and it was decided to optimize the plan on a PTV excluding the heart. | |
| **Patient 9 (3 × 8 Gy)** |  |  | **Patient 10 (3 × 20 Gy)** |  |
| **DVH parameter** | **Value** |  | **DVH parameter** | **Value** |
| PTV D99% | 24.0 Gy |  | PTV D99% | 60.0 Gy |
| PTV Dmax | 28.7 Gy |  | PTV Dmax | 68.8 Gy |
| PTV Dmean | 26.7 Gy |  | PTV Dmean | 64.5 Gy |
| Liver-ITV V15Gy | 381.0 cm^3^ |  | Liver-ITV V15Gy | 448.0 cm^3^ |
| Spinal Canal Dmax | 4.7 Gy |  | Spinal Canal Dmax | 9.3 Gy |
| Kidneys V15Gy | 9.6% |  | Kidney_R V15Gy | 0.6% |
| Kidney_R V15Gy | 11.8% |  | Esophagus Dmax | 10.4 Gy |
| Esophagus Dmax | 0.6 Gy |  | Stomach Dmax | 13.1 Gy |
| Stomach Dmax | 15.9 Gy |  | Heart Dmax | 0.8 Gy |
| Heart Dmax | 0.4 Gy |  |  |  |
|  |  |  |  |  |
| **Patient 11 (3 × 8 Gy)** |  |  |  |  |
| **DVH parameter** | **Value** |  |  |  |
| PTV D99% | 24.0 Gy |  |  |  |
| PTV Dmax | 27.9 Gy |  |  |  |
| PTV Dmean | 26.0 Gy |  |  |  |
| Liver-ITV V15Gy | 327.2 cm^3^ |  |  |  |
| Spinal Canal Dmax | 4.8 Gy |  |  |  |
| Kidneys V15Gy | 2.0% |  |  |  |
| Kidney_R V15Gy | 4.2% |  |  |  |
| Stomach Dmax | 4.4 Gy |  |  |  |
